# 1,4-Dibromo-2,5-dibut­oxy­benzene

**DOI:** 10.1107/S1600536812033338

**Published:** 2012-08-11

**Authors:** Chin Hoong Teh, Muhammad Mat Salleh, Mohamed Ibrahim Mohamed Tahir, Rusli Daik, Mohammad B. Kassim

**Affiliations:** a School of Chemical Sciences & Food Technology, Faculty of Science & Technology, Universiti Kebangsaan Malaysia, 43600 Bangi, Selangor, Malaysia; b Institute of Microengineering and Nanoelectronics (IMEN), Universiti Kebangsaan Malaysia, UKM 43500 Bangi, Selangor, Malaysia; c Department of Chemistry, Faculty of Science, Universiti Putra Malaysia, 43400 UPM Serdang, Selangor, Malaysia; d Fuel Cell Institute, Universiti Kebangsaan Malaysia, 43600 Selangor, Malaysia

## Abstract

The asymmetric unit of the title compound, C_14_H_20_Br_2_O_2_, contains one half-mol­ecule located on an inversion centre. The mol­ecule is essentially planar, with a maximum deviation from the best plane of the non-H atoms of 0.054 (2) Å for the O atoms. The but­oxy group adopts a fully extended all-*trans* conformation. In the crystal, mol­ecules are connected *via* C—Br⋯O halogen bonds [Br⋯O = 3.2393 (19) Å] into a two-dimensional corrugated network in the *bc* plane.

## Related literature

For related structures, see: Choi *et al.* (2010 ▶); Fun *et al.* (2010 ▶); Li *et al.* (2008 ▶). For applications of dialk­oxy­benzenes, see: Brandon *et al.* (1997 ▶); Huang *et al.* (2007 ▶); Lightowler & Hird (2005 ▶); Promarak & Ruchirawat (2007 ▶). For the synthetic procedure, see: Lopez-Alvarado *et al.* (2002 ▶).
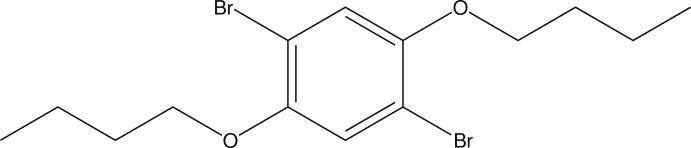


## Experimental

### 

#### Crystal data

C_14_H_20_Br_2_O_2_
*M* *_r_* = 380.10
Monoclinic, 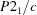

*a* = 8.3685 (4) Å
*b* = 12.6395 (5) Å
*c* = 7.1083 (3) Å
β = 96.461 (5)°
*V* = 747.10 (6) Å^3^
*Z* = 2
Cu *K*α radiation
μ = 6.82 mm^−1^
*T* = 150 K
0.07 × 0.06 × 0.01 mm

#### Data collection

Oxford Diffraction Gemini diffractometer
Absorption correction: multi-scan (*CrysAlis RED*; Oxford Diffraction, 2006 ▶) *T*_min_ = 0.647, *T*_max_ = 0.935
5426 measured reflections
1442 independent reflections
1303 reflections with *I* > 2σ(*I*)
*R*_int_ = 0.029

#### Refinement

*R*[*F* ^2^ > 2σ(*F* ^2^)] = 0.030
*wR*(*F* ^2^) = 0.081
*S* = 1.07
1442 reflections
83 parameters
H-atom parameters constrained
Δρ_max_ = 0.73 e Å^−3^
Δρ_min_ = −0.38 e Å^−3^

### 

Data collection: *CrysAlis CCD* (Oxford Diffraction, 2006 ▶); cell refinement: *CrysAlis CCD*; data reduction: *CrysAlis RED* (Oxford Diffraction, 2006 ▶); program(s) used to solve structure: *SHELXS97* (Sheldrick, 2008 ▶); program(s) used to refine structure: *SHELXL97* (Sheldrick, 2008 ▶); molecular graphics: *SHELXTL* (Sheldrick, 2008 ▶); software used to prepare material for publication: *SHELXTL*, *PLATON* (Spek, 2009 ▶) and *publCIF* (Westrip, 2010 ▶).

## Supplementary Material

Crystal structure: contains datablock(s) I, global. DOI: 10.1107/S1600536812033338/gk2508sup1.cif

Structure factors: contains datablock(s) I. DOI: 10.1107/S1600536812033338/gk2508Isup2.hkl

Supplementary material file. DOI: 10.1107/S1600536812033338/gk2508Isup3.cml

Additional supplementary materials:  crystallographic information; 3D view; checkCIF report

## Figures and Tables

**Fig. 1.**
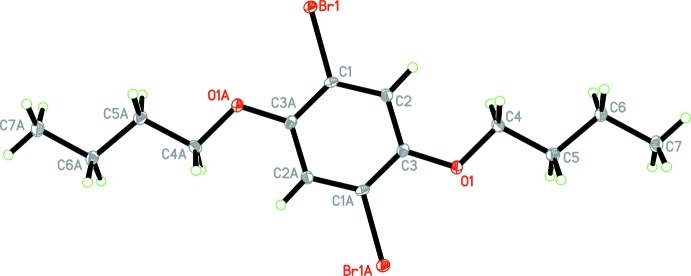
The molecular structure of the title compound, with displacement ellipsoids drawn at the 30% probability level. Symmetry code for atoms with the A label: -x, 1 - y, 1 - z.

**Fig. 2.**
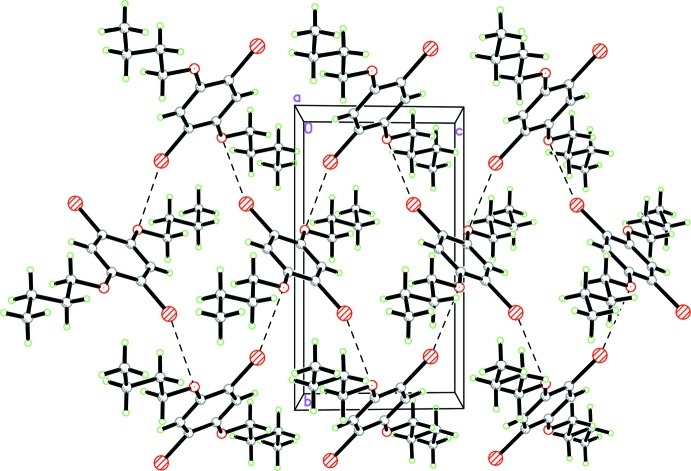
Crystal packing of the title compound showing intermolecular halogen bonds C1–Br1···O1 [-x,1/2 + y,1/2 - z] resulting in the formation of two-dimensional network along bc plane.

## supplementary crystallographic information

### Comment

Dialkoxy-substituted benzenes such as the title compound (I) are very useful intermediates to synthesize soluble poly(*p*-phenylene) (Huang *et al.*, 2007; Lightowler & Hird, 2005), thiophene–phenylene co-oligomers (Promarak & Ruchirawat, 2007) and poly(phenylene vinylene) (Brandon *et al.*, 1997), which have wide range of applications in semiconductor and electronics industries.

The title compound is similar to its analog, 1,4-dibromo-2,5-bis(hexyloxy)-benzene (II) (Li *et al.*, 2008). The alkyl chains are nearly coplanar with the benzene ring, with C4—O1—C3—C2 torsion angles of 3.3 (4)°, which is similar to II. However, the title compound is stabilized by intermolecular Br···O interactions [3.2393 (19) Å], which has shorter distance, compared to Br···Br interactions (3.410 Å) found in II. The intermolecular Br···O interaction is shorter than the sum of the Van der Waals radii of the relevant atoms (3.37 Å) and those found in other compound [3.301 (4) Å] (Fun *et al.* 2010).

In the crystal, nearly linear halogen bond C1–Br1···O1(-*x*, 1/2 + *y*, 1/2 - *z*) [<C1-Br···O159.96 (9)°] link the molecules into a two-dimensional corrugated network along *bc* plane (Figure 2).

### Experimental

The compound was prepared according to previously published work with a slight modification (Lopez-Alvarado *et al.*, 2002). To 1,4-bis(butoxy)benzene (5.00 g, 22.5 mmol) was added dropwise Br_2_ (7. 55 g, 47.25 mmol) in glacial acetic acid. The mixture was stirred at room temperature for two hours followed by heating under reflux for another two hours. The mixture was left to cool to room temperature and water was then added to precipitate the product. The product was filtered, washed with excess water and 1.0 *M* sodium bicarbonate solution. Slow recrystallization of the product from methanol–ethyl acetate mixture afforded crystals suitable for single X-ray diffraction (yield: 82%).

### Refinement

The hydrogen atom positions were calculated geometrically and refined in a riding model approximation with C–H bond lengths in the range 0.93–0.97 Å and *U*_iso_(H) = 1.2*U*_eq_(C) for aromatic and CH_2_ group, and *U*_iso_(H) = 1.5*U*_eq_(C) for methyl group.

### Crystal data

**Table:**

C_14_H_20_Br_2_O_2_	*F*(000) = 380
*M**_r_* = 380.10	*D*_x_ = 1.690 Mg m^−^^3^
Monoclinic, *P*2_1_/*c*	Melting point = 343–345 K
Hall symbol: -P 2ybc	Cu *K*α radiation, λ = 1.54178 Å
*a* = 8.3685 (4) Å	Cell parameters from 2679 reflections
*b* = 12.6395 (5) Å	θ = 3–71°
*c* = 7.1083 (3) Å	µ = 6.82 mm^−^^1^
β = 96.461 (5)°	*T* = 150 K
*V* = 747.10 (6) Å^3^	Plate, colourless
*Z* = 2	0.07 × 0.06 × 0.01 mm

### Data collection

**Table:**

Oxford Diffraction Gemini diffractometer	1442 independent reflections
Radiation source: fine-focus sealed tube	1303 reflections with *I* > 2σ(*I*)
Graphite monochromator	*R*_int_ = 0.029
ω scans	θ_max_ = 71.6°, θ_min_ = 5.3°
Absorption correction: multi-scan (*CrysAlis RED*; Oxford Diffraction, 2006)	*h* = −8→10
*T*_min_ = 0.647, *T*_max_ = 0.935	*k* = −15→15
5426 measured reflections	*l* = −8→6

### Refinement

**Table:**

Refinement on *F*^2^	Primary atom site location: structure-invariant direct methods
Least-squares matrix: full	Secondary atom site location: difference Fourier map
*R*[*F*^2^ > 2σ(*F*^2^)] = 0.030	Hydrogen site location: inferred from neighbouring sites
*wR*(*F*^2^) = 0.081	H-atom parameters constrained
*S* = 1.07	*w* = 1/[σ^2^(*F*_o_^2^) + (0.0507*P*)^2^ + 0.4756*P*] where *P* = (*F*_o_^2^ + 2*F*_c_^2^)/3
1442 reflections	(Δ/σ)_max_ < 0.001
83 parameters	Δρ_max_ = 0.73 e Å^−^^3^
0 restraints	Δρ_min_ = −0.38 e Å^−^^3^

### Special details

**Table:**

Experimental. The crystal was placed in the cold stream of an Oxford Cryosystems open-flow nitrogen cryostat (Cosier & Glazer 1986) with a nominal stability of 0.1 K.Cosier, J. & Glazer, A.*M*., (1986)., *J. Appl. Cryst.*105 107.
Geometry. All e.s.d.'s (except the e.s.d. in the dihedral angle between two l.s. planes) are estimated using the full covariance matrix. The cell e.s.d.'s are taken into account individually in the estimation of e.s.d.'s in distances, angles and torsion angles; correlations between e.s.d.'s in cell parameters are only used when they are defined by crystal symmetry. An approximate (isotropic) treatment of cell e.s.d.'s is used for estimating e.s.d.'s involving l.s. planes.
Refinement. Refinement of *F*^2^ against ALL reflections. The weighted *R*-factor *wR* and goodness of fit *S* are based on *F*^2^, conventional *R*-factors *R* are based on *F*, with *F* set to zero for negative *F*^2^. The threshold expression of *F*^2^ > σ(*F*^2^) is used only for calculating *R*-factors(gt) *etc*. and is not relevant to the choice of reflections for refinement. *R*-factors based on *F*^2^ are statistically about twice as large as those based on *F*, and *R*- factors based on ALL data will be even larger.

### Fractional atomic coordinates and isotropic or equivalent isotropic displacement parameters (Å^2^)

**Table:**

	*x*	*y*	*z*	*U*_iso_*/*U*_eq_	
Br1	−0.15758 (3)	1.17923 (2)	0.20385 (4)	0.01819 (14)	
O1	0.2931 (2)	0.90585 (15)	0.4561 (3)	0.0186 (4)	
C1	−0.0674 (3)	1.0747 (2)	0.3762 (4)	0.0165 (6)	
C2	0.0787 (3)	1.0299 (2)	0.3466 (4)	0.0170 (6)	
H2	0.1301	1.0506	0.2431	0.020*	
C3	0.1490 (3)	0.9538 (2)	0.4722 (4)	0.0160 (5)	
C4	0.3733 (4)	0.9319 (2)	0.2931 (4)	0.0188 (6)	
H4A	0.4017	1.0064	0.2949	0.023*	
H4B	0.3034	0.9175	0.1776	0.023*	
C5	0.5229 (3)	0.8644 (2)	0.3018 (4)	0.0199 (6)	
H5A	0.4923	0.7904	0.2966	0.024*	
H5B	0.5883	0.8766	0.4213	0.024*	
C6	0.6222 (3)	0.8887 (2)	0.1394 (4)	0.0202 (6)	
H6A	0.5590	0.8727	0.0198	0.024*	
H6B	0.6483	0.9635	0.1403	0.024*	
C7	0.7771 (4)	0.8243 (2)	0.1556 (5)	0.0249 (7)	
H7A	0.8411	0.8413	0.2724	0.037*	
H7B	0.8363	0.8409	0.0513	0.037*	
H7C	0.7516	0.7503	0.1535	0.037*	

### Atomic displacement parameters (Å^2^)

**Table:**

	*U*^11^	*U*^22^	*U*^33^	*U*^12^	*U*^13^	*U*^23^
Br1	0.0183 (2)	0.01468 (19)	0.0219 (2)	0.00149 (10)	0.00335 (13)	0.00358 (10)
O1	0.0161 (10)	0.0183 (9)	0.0223 (10)	0.0030 (8)	0.0061 (8)	0.0029 (8)
C1	0.0200 (15)	0.0114 (12)	0.0174 (13)	0.0022 (10)	−0.0003 (10)	0.0031 (10)
C2	0.0185 (14)	0.0134 (12)	0.0200 (14)	−0.0002 (10)	0.0063 (11)	0.0002 (10)
C3	0.0149 (13)	0.0127 (12)	0.0202 (14)	0.0005 (10)	0.0012 (10)	−0.0017 (10)
C4	0.0209 (15)	0.0175 (13)	0.0188 (14)	−0.0002 (11)	0.0062 (11)	0.0010 (11)
C5	0.0193 (14)	0.0162 (13)	0.0243 (15)	0.0012 (11)	0.0036 (11)	0.0024 (11)
C6	0.0161 (14)	0.0181 (13)	0.0269 (15)	0.0005 (11)	0.0049 (11)	0.0008 (11)
C7	0.0216 (16)	0.0241 (16)	0.0305 (18)	0.0036 (12)	0.0085 (13)	−0.0015 (12)

### Geometric parameters (Å, º)

**Table:**

Br1—C1	1.900 (3)	C5—C6	1.527 (4)
O1—C3	1.366 (3)	C5—H5A	0.9700
O1—C4	1.441 (3)	C5—H5B	0.9700
C1—C2	1.384 (4)	C6—C7	1.523 (4)
C1—C3^i^	1.386 (4)	C6—H6A	0.9700
C2—C3	1.397 (4)	C6—H6B	0.9700
C2—H2	0.9300	C7—H7A	0.9600
C4—C5	1.511 (4)	C7—H7B	0.9600
C4—H4A	0.9700	C7—H7C	0.9600
C4—H4B	0.9700		
			
C3—O1—C4	117.5 (2)	C4—C5—H5A	109.2
C2—C1—C3^i^	122.2 (3)	C6—C5—H5A	109.2
C2—C1—Br1	118.7 (2)	C4—C5—H5B	109.2
C3^i^—C1—Br1	119.1 (2)	C6—C5—H5B	109.2
C1—C2—C3	120.0 (3)	H5A—C5—H5B	107.9
C1—C2—H2	120.0	C7—C6—C5	111.5 (2)
C3—C2—H2	120.0	C7—C6—H6A	109.3
O1—C3—C1^i^	117.8 (2)	C5—C6—H6A	109.3
O1—C3—C2	124.3 (3)	C7—C6—H6B	109.3
C1^i^—C3—C2	117.8 (3)	C5—C6—H6B	109.3
O1—C4—C5	107.3 (2)	H6A—C6—H6B	108.0
O1—C4—H4A	110.3	C6—C7—H7A	109.5
C5—C4—H4A	110.3	C6—C7—H7B	109.5
O1—C4—H4B	110.3	H7A—C7—H7B	109.5
C5—C4—H4B	110.3	C6—C7—H7C	109.5
H4A—C4—H4B	108.5	H7A—C7—H7C	109.5
C4—C5—C6	112.0 (2)	H7B—C7—H7C	109.5

Symmetry code: (i) −*x*, −*y*+2, −*z*+1.

## References

[bb1] Brandon, K. L., Bentley, P. G., Bradley, D. D. C. & Dunmur, D. A. (1997). *Synth. Met.* **91**, 305–306.

[bb2] Choi, H. D., Seo, P. J., Son, B. W. & Lee, U. (2010). *Acta Cryst.* E**66**, o1042. 10.1107/S1600536810011906 PMC2979252 21579103

[bb3] Fun, H.-K., Goh, J. H., Rai, S., Isloor, A. M. & Shetty, P. (2010). *Acta Cryst.* E**66**, o1871. 10.1107/S1600536810024724 PMC3006963 21588067

[bb4] Huang, S.-P., Huang, G.-S. & Chen, S.-A. (2007). *Synth. Met.* **157**, 863–871.

[bb5] Li, Y.-F., Xu, C., Cen, F.-F., Wang, Z.-Q. & Zhang, Y.-Q. (2008). *Acta Cryst.* E**64**, o1930. 10.1107/S1600536808028730 PMC2959468 21201138

[bb6] Lightowler, S. & Hird, M. (2005). *Chem. Mater.* **17**, 5538–5549.

[bb7] Lopez-Alvarado, P., Avendano, C. & Menendez, J. C. (2002). *Synth. Commun.* **32**, 3233–3239.

[bb8] Oxford Diffraction (2006). *CrysAlis CCD* and *CrysAlis RED* Oxford Diffraction, Abingdon, England.

[bb9] Promarak, V. & Ruchirawat, S. (2007). *Tetrahedron*, **63**, 1602–1609.

[bb10] Sheldrick, G. M. (2008). *Acta Cryst.* A**64**, 112–122. 10.1107/S0108767307043930 18156677

[bb11] Spek, A. L. (2009). *Acta Cryst.* D**65**, 148–155. 10.1107/S090744490804362X PMC2631630 19171970

[bb12] Westrip, S. P. (2010). *J. Appl. Cryst.* **43**, 920–925.

